# Water and salt migration model of solidified sulfate-contaminated soil by full solid waste cementitious materials

**DOI:** 10.1371/journal.pone.0351883

**Published:** 2026-09-08

**Authors:** Chongyang Wang, Zeshi Ren, Jingwei Gong, Miaomiao Gong, Chong Shi, Zhiwei Xie, Tianqi Tong, Zunqing Liu, Jie Liu

**Affiliations:** 1 College of Hydraulic and Civil Engineering, Xinjiang Agricultural University, Urumqi, China; 2 Xinjiang Key Laboratory of Hydraulic Engineering Security and Water Disasters Prevention, Urumqi, China; 3 Geotechnical Research Institute, Hohai University, Nanjing, Jiangsu, China; 4 College of Transportation and Logistics Engineering, Xinjiang Agricultural University, Urumqi, Xinjiang, China; 5 Xinjiang Transportation Planning, Survey and Design Institute Co., Ltd., Urumqi, Xinjiang, China; Graphic Era Deemed to be University, INDIA

## Abstract

The limitations of the research on the water-salt migration of sulfate-stained soil using solid waste as the sole binding material, such as the difficulty in characterizing the hydration reaction products over time, were addressed in this study. The research focused on different ratios of solid waste-based binding materials for solidifying sulfate-stained soil. Through the combination of experiments and theory, a water-salt migration model for solidified sulfate-stained soil using solid waste-based binding materials was established. Compared to traditional studies, this model can represent the time-dependent characterization of the hydration process. By introducing the grey correlation degree theory and using porosity as the evaluation index, the dynamic evolution characterization of the hydration products of solid waste-based binding materials was achieved. An innovative approach was adopted to construct the relationship between porosity and matrix suction using the Logistic curve, which served as a link to establish the water-salt migration equation. The porosity was used to control the spatial-temporal distribution of water and salt, compensating for the traditional model's neglect of the influence of changes in the cementing system on water-salt migration. The influence of the amount of binder, the proportion of slag, and the salt content on the pore structure of the solidified soil was systematically clarified, verifying that an increase in binder amount, an increase in the proportion of slag, and a decrease in salt content can significantly optimize the pore structure and inhibit water-salt migration. This provides a theoretical basis for the water-salt migration of solidified sulfate-stained soil using solid waste-based binding materials.

## 1. Introduction

Saline soil is distributed in Europe, Asia, Africa and America, covering an area of approximately 6.5% of the global land. Xinjiang in China is one of the regions with the most extensive inland saline soil distribution, with an area of 151,830 square kilometers, The main salt type is sulfate [1, 2]. Due to their unfavorable geotechnical properties, they are often regarded as an unsuitable soil foundation materials in infrastructure projects, leading to serious engineering issues such as salt swelling, freeze-thaw expansion, slush softening, and ground subsidence in road and railway construction [3–5]. To ensure the engineering safety of saline soil areas, some studies have adopted cement to solidify it, improving its strength, impermeability, and resistance to salt heaving and freezing. However, the cement production process consumes a large amount of energy and emits a significant amount of CO_2_, causing environmental pollution. Therefore, the research on the solidification of sulfate saline soil with green building materials has become a key research area of common concern both at home and abroad.

In 2025, the output of bulk industrial solid waste (such as calcium carbide slag, fly ash and slag) in China will be approximately 4.47 billion tons, with an utilization rate of less than 30% [6, 7]. These wastes occupy a large amount of land and cause environmental problems due to their high alkalinity. These wastes contain active oxides or are alkaline and can be used to solidify sulfate soil, achieving good results in improving strength and other aspects. Numerous research results indicate that the engineering performance of saline soil is significantly related to the accumulation and migration of water and salts [8, 9]. Therefore, studying the water-salt migration laws of saline soil is a key scientific issue that needs to be addressed to comprehensively understand its engineering performance.

The current research in this field mainly focuses on conducting experiments to study the water-salt migration laws of sulfate-stained soil, and constructing water-salt migration models, thereby revealing the water-salt migration mechanism of sulfate-stained soil [10, 11]. Current water-salt migration models primarily fall into three categories: water and salt balance models, physical models, and statistical models [12]. Water-salt balance models, physical models, and statistical models. Specifically, water-salt balance models (e.g., SaltMod, SahysMod, and DrainMod) [13–15] are computationally simple but fail to capture dynamic system processes. Statistical models (e.g., fuzzy neural networks, self-organising radial basis function networks, fast backpropagation networks) [16] are easy to operate and yield good prediction accuracy. However, they require long construction periods and are limited in application range. Physical models, which originated from Darcy’s law, account for the chemical interactions between different solute components in soil salts. scholars have improved the original convection-diffusion equation (CDE) [17]. However, this equation is complex and only considers the physical and dynamic properties of solutes. To better capture solute transport under different parameter conditions, some scholars have developed models based on solute suction coefficient equations and variable-parameter transport formulations, thereby advancing study of capillary water migration [18, 19]. Using the relationship between water content and matrix potential, scholars have derived models such as Hydrus, Swap, and Shaw [20–23]. However, the above models fail to reflect the dynamic process of water and salt migration, their construction relies on long-term monitoring data, and they adopt average porosity parameters, thereby ignoring the controlling role of pore structure on water and salt migration. Compared with existing models, the model established in this paper retains the mechanism analysis ability of the physical model, while overcoming its simplification assumption of porosity. It avoids the data dependence of statistical models and can more realistically simulate the dynamic behavior of water and salt in the soil.

Traditional models of water-salt migration in saline soils are largely built on Darcy’s law, with emphasis on solute transport, moisture content, and matric suction, providing important references for the utilization of saline soil.Current studies on the water-salt migration of stabilized saline soil primarily focus on spatiotemporal distribution patterns of water and salt [24], often neglecting the dynamic filling of soil pores by hydration products of all-solid-waste cementitious materials. Furthermore, the water-salt migration model is limited by the difficulty in constructing accurate relationships between matric suction and the aging of hydration products. The research on the solidification of sulfate-stained soil using all-solid waste cementitious materials mainly focuses on the optimization of mechanical properties: through experiments, the influence of different cementitious material ratios on the unconfined compressive strength is investigated, or a strength prediction model based on projection pursuit regression is established [25, 26]. The traditional water-salt migration models are mostly based on soil, a single and multiple solidification materials, but they do not consider the complexity of the hydration products of the all-solid waste system, and lack coupling modeling of indicators such as porosity and matrix suction [27, 28].

In conclusion, there are few reports on the water-salt migration model for sulfate saline soils stabilized with all-solid-waste cementitious materials. Therefore, this study takes all-solid-waste cementitious materials composed of calcium carbide slag (C), fly ash (F), and slag (S) as the research object. Through water-salt migration experiments, the basic indicators such as matric suction, moisture content, porosity, and salt content are measured to explore the migration characteristics and governing laws. On this basis, pore porosity is used to characterize the time-dependent evolution of hydration products, and a logistic curve is used to construct the relationship between pore porosity and matric suction. By coupling the water migration and salt migration equations, a coupled model for water-salt migration in sulfate saline soil is established. Further, through decoupling analysis, the calculation accuracy of this model is verified. Finally, based on this model, the influence of pore porosity on water-salt migration and the model’s applicability are analyzed. The effects of the composition of cementitious materials under high-, medium-, and low- salinity conditions are also discussed, providing a theoretical basis for the engineering application of all-solid-waste cementitious materials in sulfate saline soil.

## 2. Theoretical analysis basis

### 2.1 Water migration control equation

Water migration within sulfate saline soil stabilized with all-solid-waste cementitious material follows Darcy’s law. Since soil water is considered incompressible, its density is assumed constant [29]. According to the moisture migration control equation established by Darcy’s law, this migration process satisfies the law of mass conservation [30]. This means that the changes in the water fluxes flowing into and out of the infinitesimal volume along the x, y, and z directions are as follows:


$$\begin{array}{c} q_{x}+\frac{\partial q_{x}}{\partial x}\Delta x-q_{x}=\frac{\partial q_{x}}{\partial x}\Delta x \end{array}$$ (1)

$$\begin{array}{c} q_{y}+\frac{\partial q_{y}}{\partial y}\Delta y-q_{y}=\frac{\partial q_{y}}{\partial y}\Delta y \end{array}$$ (2)

$$\begin{array}{c} q_{z}+\frac{\partial q_{z}}{\partial z}\Delta z-q_{z}=\frac{\partial q_{z}}{\partial z}\Delta z \end{array}$$ (3)

Where: *q*_*x*_, *q*_*y*_,*q*_*z*_ represent the fluxes in the x, y, and z directions, respectively, m/s.

During the time interval Δt, the changes in water flux of the infinitesimal element in the x, y, and z directions are as follows:


$$\begin{array}{c} \frac{\partial \left(q_{x}\rho_{w}\right)}{\partial x}\Delta x\Delta y\Delta z\Delta t \end{array}$$ (4)

$$\begin{array}{c} \frac{\partial \left(q_{y}\rho_{w}\right)}{\partial y}\Delta x\Delta y\Delta z\Delta t \end{array}$$ (5)

$$\begin{array}{c} \frac{\partial \left(q_{z}\rho_{w}\right)}{\partial z}\Delta x\Delta y\Delta z\Delta t \end{array}$$ (6)

Where: ρ_w_ is the density, kg/m³.

The variation in the flow of liquid water within the micro-element is as follows:


$$\begin{array}{c} \Delta m=\Delta m_{x}+\Delta m_{y}+\Delta m_{z}=-\left[\frac{\partial \left(q_{x}\rho_{w}\right)}{\partial x}+\frac{\partial \left(q_{y}\rho_{w}\right)}{\partial y}+\frac{\partial \left(q_{z}\rho_{w}\right)}{\partial z}\right]\Delta x\Delta y\Delta z\Delta \end{array}$$ (7)

Where:Δm represents the increment of water content within the infinitesimal element (g), while Δmx, Δmy, and Δmz respectively denote the increments of water content in the three coordinate directions of the infinitesimal element (g). By simplifying the calculation using the Hamiltonian operator ▽, the above equation can be written as:


$$\begin{array}{c} \Delta m=-\nabla \left(\rho_{w}q\right)\Delta x\Delta y\Delta z\Delta t \end{array}$$ (8)

During the time interval Δt, the mass of water in the microelements within the consolidated soil mass increased by Δm:


$$\begin{array}{c} \Delta m=\frac{\partial \left(\rho_{w}\theta \right)}{\partial t}\Delta x\Delta y\Delta z\Delta t \end{array}$$ (9)

Therefore, In Δt time, the increase in water content within the infinitesimal body is equal to the difference in moisture mass flowing into and out of the infinitesimal body, as shown in Eq. (10):


$$\begin{array}{c} \frac{\partial \left(\rho_{w}\theta \right)}{\partial t}=-[\frac{\partial \left(\rho_{w}q_{x}\right)}{x}+\frac{\partial \left(\rho_{w}q_{y}\right)}{y}+\frac{\partial \left(\rho_{w}q_{z}\right)}{z}] \end{array}$$ (10)

Where: θ is the volumetric water content, %; q is the water flux.

According to Darcy’s law, the relationship between the water flux q in the soil and the soil water potential ϕ is given by Eq. (11):


$$\begin{array}{c} q=-k\nabla \varphi =-k\left(\frac{\partial \varphi_{x}}{\partial x}+\frac{\partial \varphi_{y}}{\partial y}+\frac{\partial \varphi_{\text{z}}}{\partial \text{z}}\right) \end{array}$$ (11)

Where, k is the permeability coefficient.

Since water is incompressible and density remains constant [31], substituting Eq. (11) into Eq. (10) yields the equation for moisture migration:


$$\begin{array}{c} \frac{\partial \theta }{\partial t}=k(\frac{\partial^{\text{2}}\varphi_{x}}{\partial x^{\text{2}}}+\frac{\partial^{\text{2}}\varphi_{y}}{\partial y^{\text{2}}}+\frac{\partial^{\text{2}}\varphi_{z}}{\partial z^{\text{2}}}) \end{array}$$ (12)

In soil, water migration occurs primarily in liquid form, and vapor-phase transport can be neglected [32]. And the fully solidified waste cementitious materials used to solidify sulfate-stained soil for roadbed projects have extremely low permeability in the horizontal direction, resulting in the horizontal water migration volume being much smaller than the vertical direction [33]. Therefore, the horizontal migration volume can be ignored, and thus a one-dimensional mathematical model can be simplified:


$$\begin{array}{c} \frac{\partial \theta }{\partial t}=k\frac{\partial^{\text{2}}\varphi_{z}}{\partial z^{\text{2}}} \end{array}$$ (13)

Soil water potential represents the potential energy of water in the soil and reflects the potential energy difference between any two points in the soil. It is the driving force for water migration. Soil water potential consists of the base water potential (ϕ_m_), gravitational potential (ϕ_g_), solute potential (ϕ_s_), temperature potential (ϕ_r_), and pressure potential (ϕ_p_). Among them, gravitational potential is commonly referred to as the elevation head, expressed as z [34]. When the solid waste cementitious materials are used to solidify the sulfate-stained soil, the solute concentration is uniformly distributed, the internal pressure changes are extremely small, and the temperature variation range during the experiment is also small. This makes the effects of solute potential, pressure potential and temperature potential on water migration negligible [35]. Therefore, the soil-water potential is expressed as:


$$\begin{array}{c} \varphi =\varphi_{g}+\varphi_{m}+\varphi_{\text{s}}+\varphi_{\text{r}}+\varphi_{\text{p}}=\varphi_{g}+\varphi_{m}=z+\varphi_{m} \end{array}$$ (14)

### 2.2 Salt transport control equation

The total flux of salt solutes in soil (J) is the sum of the convective flux (J_C_) and the hydrodynamic dispersion flux (J_sh_) [36]. The one-dimensional governing equation for salt migration can be expressed as:


$$\begin{array}{c} J=J_{C}+J_{sh}=qc-D_{sh}\frac{\partial c}{\partial z} \end{array}$$ (15)

Where q is the represents the volume flux of water, c is the solute concentration, g/kg; D_sh_ is the hydrodynamic dispersion coefficient; z is the direction of salt migration.

The movement of water carries salt with it, and the direction and speed of water flow directly determine the path and speed of salt convection migration. The velocity gradient generated by water flow will trigger hydrodynamic dispersion, further promoting the diffusion of salt along the concentration gradient direction. Together, these two processes complete the overall migration of salt.

Salt transport in soil also satisfies the law of mass conservation, which can be expressed as:


$$\begin{array}{c} \frac{\partial \left(\theta c\right)}{\partial t}=-\frac{\partial \left(q_{x}c\right)}{\partial x}-\frac{\partial \left(q_{y}c\right)}{\partial y}-\frac{\partial \left(q_{z}c\right)}{\partial z}+\frac{\partial }{\partial x}\left(D_{sh}\frac{\partial c}{\partial x}\right)+\frac{\partial }{\partial y}\left(D_{sh}\frac{\partial c}{\partial y}\right)+\frac{\partial }{\partial z}\left(D_{sh}\frac{\partial c}{\partial z}\right) \end{array}$$ (16)

$\frac{\partial \left(\theta c\right)}{\partial t}$ represents the rate of change of salt concentration over time. The first term on the right side is the flux change term of salt carried by water flow $\frac{\partial \left(q_{x}c\right)}{\partial x}$, and the second term is the hydrodynamic dispersion term $\frac{\partial }{\partial x}\left(D_{sh}\frac{\partial c}{\partial x}\right)$. This indicates that the change in water flow directly affects the rate of salt migration, and at the same time, the change in salt concentration distribution will also interact with water flow through influencing its flow characteristics, forming a dynamic coupling relationship.

Since salt transport in soil primarily occurs vertically, the above equation can be simplified into a one-dimensional model:


$$\begin{array}{c} \frac{\partial \left(\theta c\right)}{\partial t}=-\frac{\partial \left(q_{z}c\right)}{\partial z}+\frac{\partial }{\partial z}\left(D_{sh}\frac{\partial c}{\partial z}\right) \end{array}$$ (17)

The one-dimensional formula in the z-direction is:


$$\begin{array}{c} q=-k\nabla \varphi =-k\frac{\partial \varphi_{\text{z}}}{\partial \text{z}} \end{array}$$ (18)

Where k is the permeability coefficient.

From Eq. (17) and (18), it can be seen that the flow velocity q_z_ of water is determined by the water head gradient, which directly determines the intensity of salt convection migration; while the dispersion coefficient D_sh_ generated by water flow also affects the diffusion range of salt, and the two together determine the migration law of salt in the vertical direction.

## 3. Test scheme and results

### 3.1 Test materials and scheme

#### 3.1.1 Test materials.

The basic physical properties of the test soil are summarized in Table 1. The all-solid-waste cementitious materials used include calcium carbide slag (C), fly ash (F), and slag (S). Their chemical compositions are listed in Table 2. Distilled water was used in the tests, and anhydrous sodium sulfate with a purity of 99% was selected as the saline regulator.

**Table 1:** Basic physical properties of the test soil samples.

Liquid limit /%	Plastic limit /%	Salinity /%	Plasticity index	Optimum moisture content /%	Maximum dry density/ (g×cm^–3^)
28.35	16.68	0.6	11.67	12.91	1.93

**Table 2:** Main chemical composition of cementitious materials.

Material	Chemical composition							
	SiO_2_	Al_2_O_3_	Fe_2_O_3_	CaO	MgO	SO_3_	K_2_O	Na_2_O
**C**	4.71	1.36	0.85	69.6	0	0.1	0.03	0.06
**F**	56.17	20.96	6.05	5.86	2.49	1.13	2.66	1.04
**S**	37.94	18.43	0.92	39.00	2.14	0.22	0.11	0.11

#### 3.1.2 Test scheme.

Previous research has shown that when calcium carbide slag accounts for 15% of the total cementitious material is 15%, it provides effective alkaline activation. Therefore, the proportion of calcium carbide slag was set at 15% [25]. According to the salt content, saline soil is classified into low-salinity soils (0.3%–2%), medium-salinity soils (2%–5%), and high-salinity soils (>5%) [25, 37]. To investigate the effects of salt content, cementitious material composition (i.e., the mix proportion of calcium carbide slag, fly ash, and slag), and cementitious material dosage on water-salt migration, four salinity levels (0.3%, 1%, 2%, and 3%) were selected for analysis. According to the Technical Specifications for Road Pavement Base Construction, the 7-day unconfined compressive strength of stable base materials for secondary and lower-grade roads must be ≥ 0.5 MPa. Following the recommendations of [38], the dosage of cementitious materials was kept below 8%. Four dosages of cementitious material were tested: 2%, 4%, 6%, and 8%, each of which satisfied the above requirements, as shown in Table 1. Three systems with varying mix proportions of slag and fly ash were tested: the S system (85% slag), the F + S system (42.5 fly ash + 42.5% slag), and the F system (85% fly ash). The detailed test scheme is presented in Table 3.

**Table 3:** Test scheme.

Group	Test No.	J/%	T/%	C/%	S/%	F/%	N/%	Height
Computing Group	A1	2	98	15	85	0	0.3	10
	A2	2	98	15	85	0	1	
	A3	2	98	15	85	0	2	
	A4	2	98	15	85	0	5	
	A5	2	98	15	42.5	42.5	2	
	A6	2	98	15	0	85	2	
	A7	4	96	15	85	0	2	
	A8	6	94	15	85	0	2	
	A9	8	92	15	85	0	2	
Verification Group	Y1	6	94	15	50	50	1	
	Y2	6	94	15	50	50	2	

Note: J = cementitious material dosage; T = soil dosage; C = calcium carbide slag dosage; S = slag dosage; F = fly ash dosage; N = sulfate content. The proportions of calcium carbide slag, slag, and fly ash are expressed as percentages of the cementitious material dosage.

**Fig 1:**
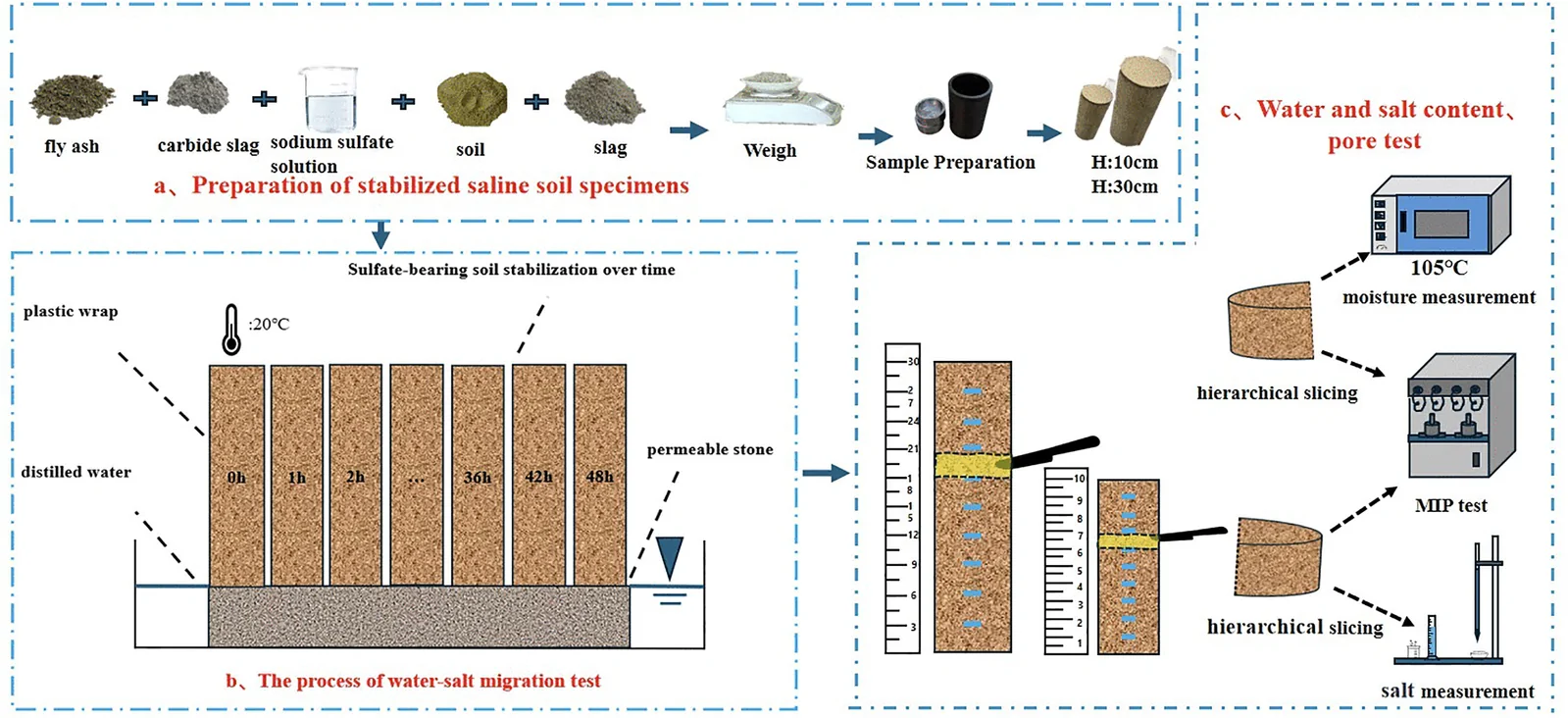
Test flowchart.

The experimental process is illustrated in Fig 1. The specimen preparation followed the Standard for Geotechnical Testing Methods [39], as shown in Fig 1(a). The mixture was compacted in three layers, with 25 blows per layer, to a target compaction degree of 98%. The water content and dry density were maintained at 1.86% and 13.24 g·cm^−^³, respectively. Cylindrical specimens with dimensions of Ф61.8 mm × H300 mm were then sealed and cured in a standard curing box for 7 days prior to testing for the water-salt migration test.

Continuous water supply was applied at the bottom of the specimen to simulate natural water migration from a subgrade base (Fig 1(b)). To minimize temperature-related interference, the ambient temperature was kept at 20 ℃. A plastic film was wrapped around the solidified saline soil to prevent water loss from the surrounding soil. the water-salt migration was monitored and recorded at 0, 1, 2, 3, 6, and 24 hours.

In Fig 1(c), to analyze the temporal and spatial distribution pattern of water and salt migration in the test specimens, for each time point of the specimens, the specimens were sectioned at intervals of 1 cm. The sections were divided into three parts. One part was tested for moisture content using the drying method, with the drying temperature set at 105℃ ± 2℃, and the drying time not less than 8 hours. The drying process was continued until a constant weight was achieved. Weighing was performed using an electronic balance with an accuracy of 0.01g. Each parallel sample was measured twice, and the average value was taken as the final result. The relative standard deviation (RSD) was ≤ 2%. Another part was tested for sulfate ions using the EDTA indirect titration method [40]. Each sample was injected three times, and the relative standard deviation (RSD) was ≤ 3%. The porosity of each sample was tested using a mercury porosimeter, and the test was repeated twice for each sample. If the deviation between the two test results exceeded 5%, a third test was conducted, and the average value of the two results with smaller deviation was taken. All test groups had 3 parallel specimens. The test results of the parallel specimens were taken as the arithmetic mean. If the deviation between a single specimen's test result and the average value exceeded 10%, the test was repeated.

### 3.2 Test results

#### 3.2.1 Effect of cementitious material dosages on spatiotemporal distribution of water and salt migration.

**Fig 2:**
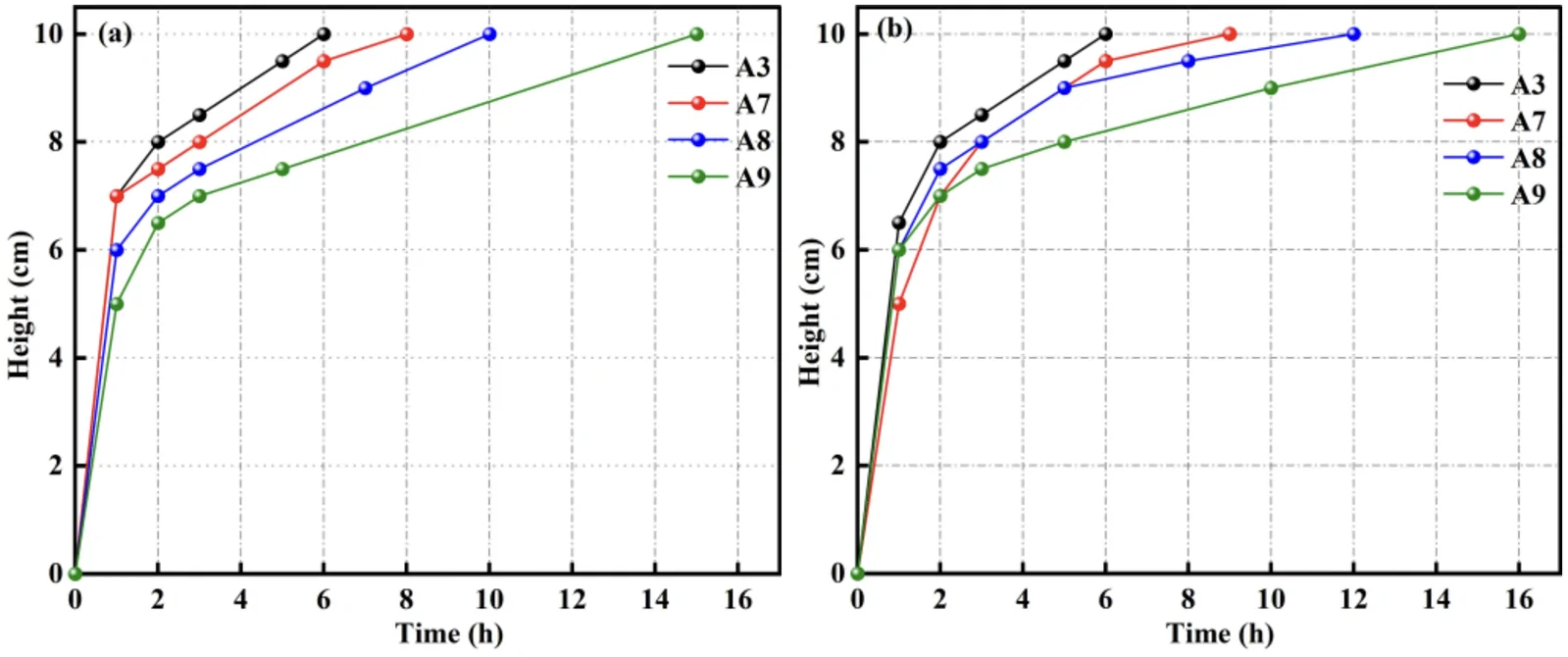
Variation in moisture and salt content for different amounts of cementitious materials: (a) water rise height, (b) salt rise height.

Using A3, A7, A8, and A9 as examples, the influence of varying amounts of cementitious materials on the temporal and spatial distribution of water-salt migration was analyzed. The changes in moisture and salt content for each specimen over time are shown in Fig 2. The water and salt migration rates exhibited a consistent pattern across all specimens: initially increased rapidly and then slowed down. As cementitious materials dosage increased, the height of water and salt migration in each period showed a downward trend.

From Fig 2(a), it can be seen that three typical time points of 0h, 2h, and 6h were selected for analysis. The initial moisture contents of different test blocks were different. Due to the fact that the test blocks absorbed moisture from the bottom and transported it upwards while being affected by factors such as gravity, the peak values of moisture accumulation at 2h for A3 and A7 were 6.8 cm and 6.5 cm respectively, while for A9 it was only 5.2 cm. As the test time continued, this accumulation phenomenon gradually increased. The average migration rates of A3, A7, and A9 within 0-6h were 1.63 cm/h, 1.53 cm/h, and 1.42 cm/h respectively, indicating that the overall moisture of A3 and A7 changed more significantly within 6h than A9. This suggests that the higher the dosage of the cementitious material, the more significant the blocking effect, and the lower the moisture migration rate. From Fig 2(b), it can be seen that the initial salt contents of different test blocks were different. Within 2h, the salt content migrated to approximately 7 cm. At 6h, due to the fact that the internal moisture of the test blocks was basically filled with channels, the salt diffused freely, resulting in significant fluctuations in the salt content at different heights. The average migration rates of A3 and A9 within 0-6h were 1.37 cm/h and 1.13 cm/h respectively. The fluctuation range of A3's salt content was 12.5%, while that of A9 was only 7.2%. This indicates that increasing the dosage of the cementitious material can reduce the salt migration rate and the volatility of salt migration.

Fig 2 demonstrates that salt migration proceeded at a lightly slower rate than water migration. Moreover, water-salt fluctuations at different heights in specimen A9 were less pronounced than those in A3 and A7. This indicates that increasing the dosage of cementitious materials provides an effective barrier within the soil, altering the diffusion behavior of both water and salt and reducing their overall migration rate.

#### 3.2.2 Effect of cementitious material composition on spatiotemporal distribution of water and salt migration.

**Fig 3:**
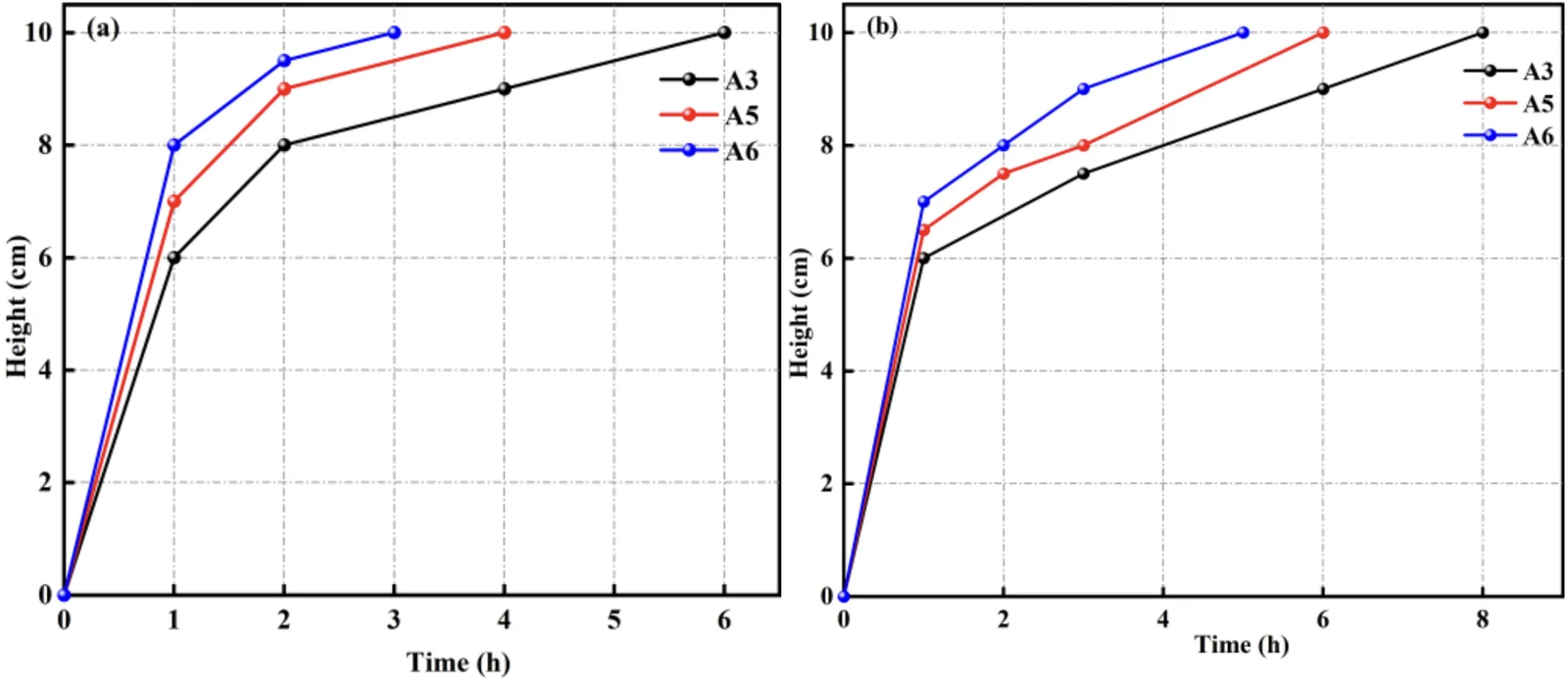
Variation in water and salt migration height over time for different mix proportions: (a) water rise height, (b) salt rise height.

Using specimens A3, A5 and A6 as examples, the influence of different cementitious material compositions (mix proportions of calcium carbide slag, slag, and fly ash) on the temporal and spatial distribution of water and salt migration was analyzed. The variations in water and salt rise height over time are shown in Fig 3. Overall, both water and salt exhibited a migration pattern characterized by a rapid initial rise followed by a gradual slowdown. The increase rate was fastest within the first hour. As the proportion of calcium carbide slag increased, the migration heights of water and salt gradually declined at each stage, while the time required for migration to reach the top of the specimen increased slightly.

As shown in Fig 3(a), the initial water content distribution at 0 h varied across different mix proportions. Within the hour, specimens absorbed water rapidly, and the migration rate was extremely high. As the rise height increased, the migration rate slowed, and by 3 h, the water rise height was limit3ed to only 2–3 cm. Among the three specimens, A3 exhibited relatively slower water migration, demonstrating a noticeable inhibitory effect. Fig 3(b) shows that during the early stage of the experiment (0–2 h), the salt rise height increased rapidly. The hydration reaction of slag is faster than that of fly ash, and it generates more hydration products such as C-S-H gel. These hydration products fill the internal pores, restricting the rapid migration of water and blocking the diffusion channels for salt. The A3 slag has the highest content and the most compact pore structure, thus the rate of salt migration is the slowest. The rate of salt migration for different ratios is from largest to smallest as follows: A6 > A5 > A3. The spatiotemporal distribution results indicate that slag-fly ash mix proportions significantly affected salt migration. At the bottom of the specimen, salt content increased markedly with time, accompanied by an upward rise in migration height.

A close relationship was observed between water and salt migration. Rapid water migration facilitated salt transport, and the magnitude of salt variation at the specimen bottom exceeded that of water. Overall, increasing the proportion of slag exerted a certain inhibitory effect on both water and salt migration.

#### 3.2.3 Effect of salt contents on spatiotemporal distribution of water and salt migration.

**Fig 4:**
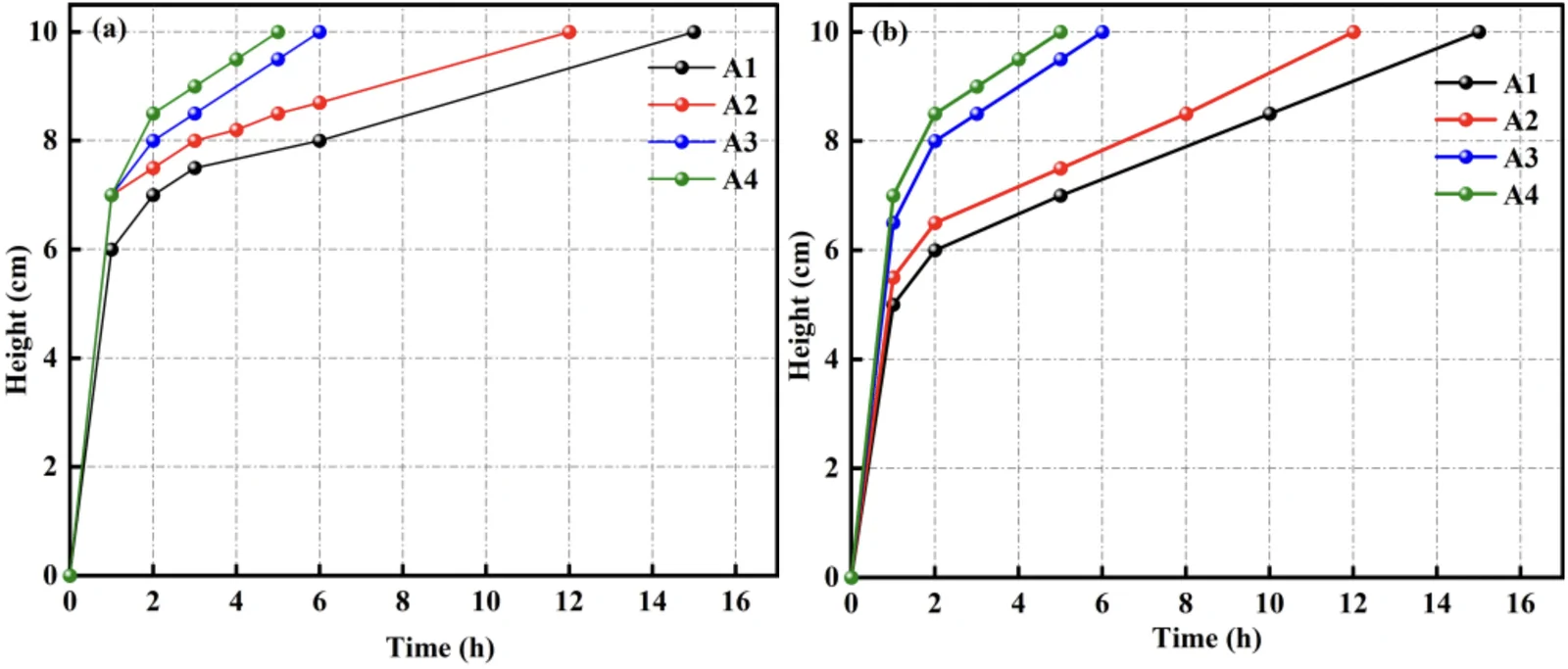
Variation in water and salt migration under different salt contents: (a) water rise height, (b) salt migration height.

Using specimens A1, A2, A3 and A4 as examples, the influence of different salt contents on the temporal and spatial distribution of water-salt migration was analyzed. The results are shown in Fig 4.

From Fig 4(a), under low-salt conditions, water migration proceeded relatively slowly, whereas under high-salt conditions, the migration rate increased markedly. For specimens A2 and A3, a distinct turning point in water migration rate was observed after 2 h. When salt concentration was lower than A2, the rate of water migration increased relatively slowly; was higher than A2, the water migration rate increased significantly.

As shown in Fig 4(b), during the initial stage, salt migration in all specimens exhibited a rapid upward trend. With time, however, the migration rates under different salt concentration conditions began to show differences. Under high-salt conditions, the migration rate was faster, while under low-salt conditions, migration was slower and dispersion effects were comparatively weaker.

## 4. Computational model for water-salt migration based on porosity effect

### 4.1 Equation for water-salt migration in sulfate saline soil treated with all-solid-waste cementitious materials

**Fig 5:**
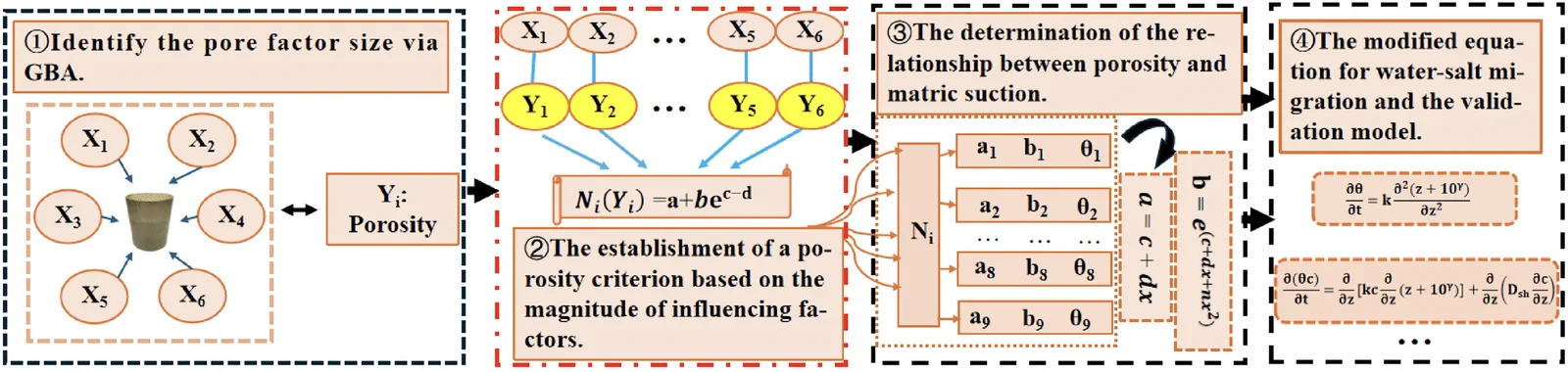
Schematic diagram of the model establishment process.

This study takes Darcy's law as the core theoretical basis, combines the hydration reaction characteristics of full solid waste cementitious materials in curing sulfate saline soil, and uses porosity as a bridge to connect the relationship between various influencing factors and water-salt migration. For six influencing factors including soil particle size (X_1_), cementitious material dosage (X_2_), calcium carbide slag dosage (X_3_), fly ash dosage (X_4_), salt content (X_5_), and curing age (X_6_), the grey relational analysis method is adopted to conduct a correlation degree analysis. This is because this method does not rely on a large amount of sample data and can measure the correlation degree between multiple factors and porosity through the geometric similarity between sequences. It is particularly suitable for the experimental scenario of multi-factor coupling and limited sample size in this study. Compared with methods such as multiple regression analysis, it makes fewer assumptions about data distribution and can more objectively reflect the nonlinear and fuzzy correlation relationships in engineering systems. After calculation, the influence degree of each factor on porosity is determined and ranked. Based on this ranking, a porosity prediction criterion for multi-factor coupling is constructed. Subsequently, based on the soil water characteristic curve theory, a quantitative relationship between porosity and matric suction is established. Finally, the porosity criterion and the matric suction model are substituted into the water-salt migration equation to obtain a modified water-salt migration model for full solid waste cementitious materials curing sulfate saline soil. The conceptual framework of the model is shown in Fig 5.

#### 4.1.1 Effect of porosity on water and salt migration.

Table 4 presents the distribution of pore characteristic parameters of stabilized sulfate saline soil under various cementitious material dosages, mix proportions of slag and fly ash, and salt contents.

**Table 4:** Distribution of pore feature parameters.

Test No.	porosity /%	Maximum pore volume /(mL⋅g^-1^)	Pore volume fraction /%		
			<600nm	[600nm,6000nm)	≥6000nm
A1	31.76	0.1845	41.2	15.9	42.9
A2	31.84	0.1801	39.6	14.5	45.9
A3	33.65	0.1931	38.8	10.9	50.3
A4	31.92	0.1851	35.7	15.8	48.5
A5	31.13	0.1773	35.1	20.4	44.4
A6	30.59	0.1712	37.1	21.6	41.3
A7	29.94	0.1679	39.0	27.6	33.4
A8	32.39	0.1885	33.3	15.9	50.8
A9	32.78	0.1891	35.1	13.3	51.5

**Fig 6:**
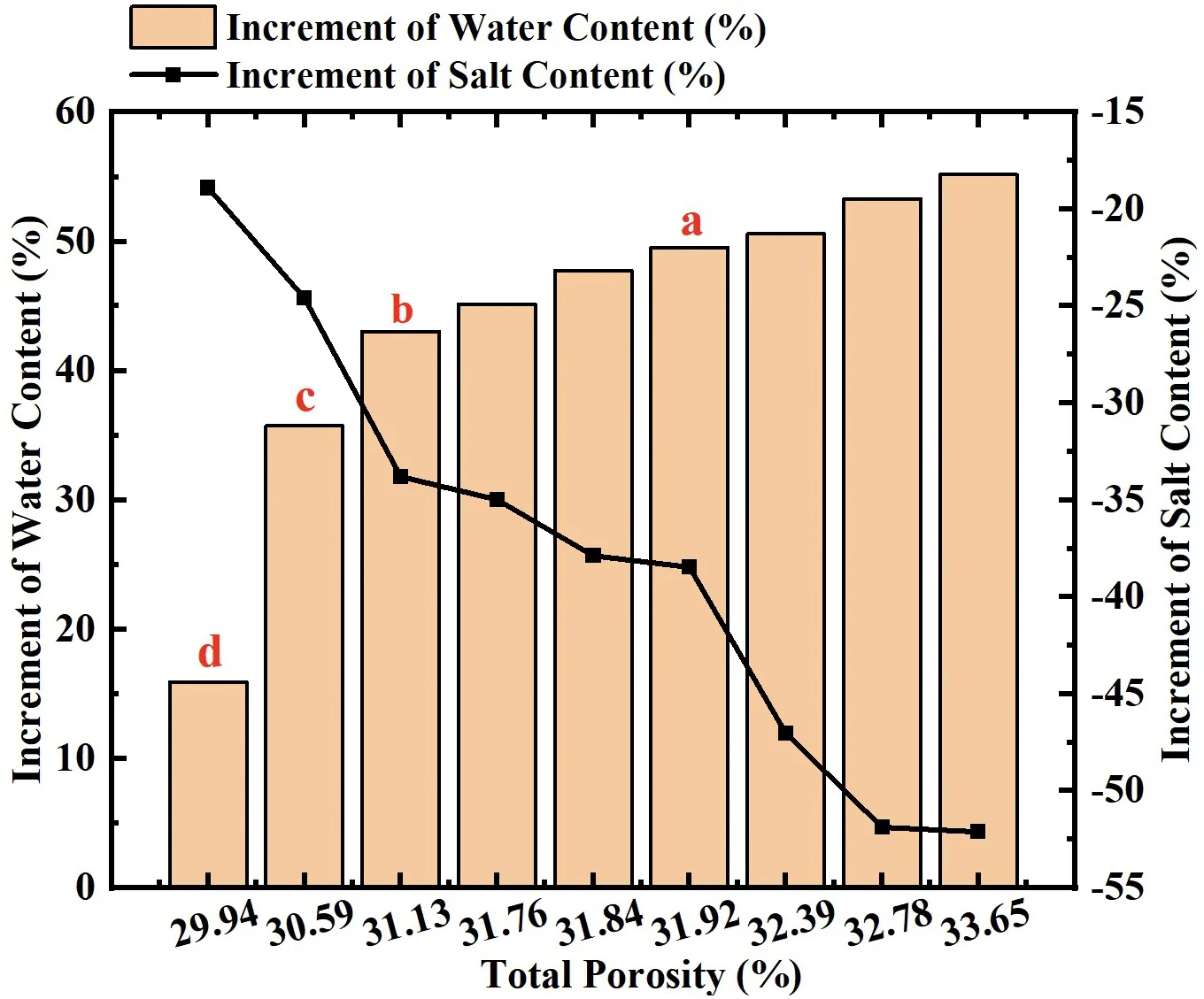
Variations in water and salt content increments with changes in soil porosity.

Fig 6 compares the increments of water content and salt content under different porosity conditions. Results show that water and salt migration generally increased higher salt content and greater fly ash dosage. The dosage of cementitious materials exerted a significant impact on water and salt migration. Its increase reduced the porosity (from 31.92% to 29.94%), and significantly reduced the increments of water migration (from 49.53% to 15.92%) and salt migration (from −18.9% to −49.53%).

#### 4.1.2 Grey correlation analysis of porosity parameters.

The major factors affecting porosity include the type and proportion of curing agents, curing age, particle size, and material type [41, 42]. Grey correlation analysis can effectively reveal the relationships among these factors and their influence on porosity. Therefore, this analysis method was applied to investigate the correlation between these influencing factors and porosity [43]. A higher grey correlation degree indicates a stronger influence of a given factor on the porosity of sulfate saline soil, whereas a lower degree implies a weaker effect [44].

(1) Determination of independent and dependent variable matrices

The independent variable matrix is formed by arranging the factors affecting porosity of the stabilized sulfate saline soil in a logical relationship [45]. The dependent variable matrix corresponds to the strength of the sulfate saline soil. The two are in a one-to-one correspondence, as expressed in Eqs. (19) and (20):


$$\begin{array}{c} X=\left[\begin{array}{c} X_{\text{1}}\\ X_{\text{2}}\\ N\\ X_{\text{m}} \end{array}\right]=\left[\begin{array}{cccc} x_{\text{1}}\left(1\right) & x_{\text{1}}\left(2\right) & \cdots & x_{\text{1}}\left(n\right)\\ x_{\text{2}}\left(1\right) & x_{\text{2}}\left(2\right) & \cdots & x_{\text{2}}\left(n\right)\\ \cdots & \cdots & \cdots & \cdots \\ x_{\text{m}}\left(1\right) & x_{\text{m}}\left(2\right) & \cdots & x_{\text{m}}\left(n\right) \end{array}\right] \end{array}$$ (19)

$$\begin{array}{c} Y=\left[\begin{array}{c} Y_{\text{1}}\\ Y_{\text{2}}\\ N\\ Y_{\text{m}} \end{array}\right]=\left[\begin{array}{cccc} {\text{y}}_{\text{1}}\left(1\right) & y_{\text{1}}\left(2\right) & \cdots & y_{\text{1}}\left(n\right)\\ y_{\text{2}}\left(1\right) & y_{\text{2}}\left(2\right) & \cdots & y_{\text{2}}\left(n\right)\\ \cdots & \cdots & \cdots & \cdots \\ y_{\text{m}}\left(1\right) & y_{\text{m}}\left(2\right) & \cdots & y_{\text{m}}\left(n\right) \end{array}\right] \end{array}$$ (20)

Where X_i_ is the factor i that affects the porosity of the sulfate saline soil; Y_i_ is the corresponding porosity; x_i(j)_ is the j-th value of factor i; y_i(j)_ is the porosity corresponding to x_i_(j).

(2) Calculation of the difference matrix

To construct the difference matrix, both independent variable and dependent variable matrices must be normalized. The normalization formulas are given in Eqs. (21)–(24):


$$\begin{array}{c} X_{i}^{{\;}^{\prime }}=\left[x_{i}^{{\;}^{\prime }}\left(1\right)\;x_{i}^{{\;}^{\prime }}\left(2\right)\cdots x_{i}^{{\;}^{\prime }}\left(n\right)\right] \end{array}$$ (21)

$$\begin{array}{c} Y_{i}^{{\;}^{\prime }}=\left[y_{i}^{{\;}^{\prime }}\left(1\right)\;y_{i}^{{\;}^{\prime }}\left(2\right)\cdots y_{i}^{{\;}^{\prime }}\left(n\right)\right] \end{array}$$ (22)

$$\begin{array}{c} {x_{i}}^{{\;}^{\prime }}\left(j\right)=\frac{x_{i}\left(j\right)-minx_{i}\left(j\right)}{maxx_{i}\left(j\right)-minx_{i}\left(j\right)} \end{array}$$ (23)

$$\begin{array}{c} {y_{i}}^{{\;}^{\prime }}\left(j\right)=\frac{y_{i}\left(j\right)-miny_{i}\left(j\right)}{maxy_{i}\left(j\right)-miny_{i}\left(j\right)} \end{array}$$ (24)

After normalization, the independent variable and dependent variable matrices are subtracted, and the absolute values of the corresponding differences are taken to form the difference matrix D, as shown in Eq. (25):


$$\begin{array}{c} D=\left[\begin{array}{cccc} D_{\text{1}}\left(1\right) & D_{\text{1}}\left(2\right) & \cdots & D_{\text{1}}\left(n\right)\\ D_{\text{2}}\left(1\right) & D_{\text{2}}\left(2\right) & \cdots & D_{\text{2}}\left(n\right)\\ \cdots & \cdots & \cdots & \cdots \\ D_{m}\left(1\right) & D_{m}\left(2\right) & \cdots & D_{m}\left(n\right) \end{array}\right] \end{array}$$ (25)

$$\begin{array}{c} D_{i}\left(j\right)=\left|{x_{i}}^{{\;}^{\prime }}\left(j\right)-{y_{i}}^{{\;}^{\prime }}\left(j\right)\right| \end{array}$$ (26)

Therefore, the grey relational degree can be calculated according to Eq. (27):


$$\begin{array}{c} \lambda_{\text{i}}=\frac{\text{1}}{m}\cdot \sum_{j=1}^{m}\eta_{ij} \end{array}$$ (27)

$$\begin{array}{c} \eta_{\text{ij}}=\frac{D_{\text{min}}+\rho D_{\text{max}}}{D_{ij}+\rho D_{max}} \end{array}$$ (28)

Where η_ij_ is the correlation coefficient; ρ is the resolution coefficient, typically set to 0.5; and D_min_ and D_max_ are the minimum and maximum values in the difference matrix, respectively.

The influence of a factor on the solidified sulfate soil is expressed by the magnitude of its grey relational degree. A higher grey relational degree indicates a stronger influence, whereas a lower degree indicates a weaker effect.

Based on the above theory and the measured data by the research group, the independent variable matrix and the dependent variable matrix are calculated, as shown in Eqs. (29) and (30).


$$\begin{array}{c} X=\left[\begin{array}{c} X_{\text{1}}\\ X_{\text{2}}\\ X_{\text{3}}\\ X_{\text{4}}\\ X_{\text{5}}\\ X_{\text{6}} \end{array}\right]=\left[\begin{array}{cccccc} 1.00 & 2.00 & 5.00 & 8.00 & 2.00 & 3.00\\ 0.00 & 1.00 & 0.00 & 3.84 & 2.34 & 1.56\\ 5.72 & 4.97 & 6.99 & 2.76 & 4.46 & 1.75\\ 4.86 & 5.77 & 3.97 & 5.96 & 1.95 & 6.96\\ 4.56 & 2.76 & 6.99 & 6.94 & 1.82 & 5.82\\ 446.65 & 442.33 & 432.12 & 330.21 & 122.63 & 431.12 \end{array}\right] \end{array}$$ (29)

$$\begin{array}{c} Y=\left[\begin{array}{c} Y_{\text{1}}\\ Y_{\text{2}}\\ Y_{\text{3}}\\ Y_{\text{4}}\\ Y_{\text{5}}\\ Y_{\text{6}} \end{array}\right]=\left[\begin{array}{cccccc} 1.00 & 2.00 & 5.00 & 8.00 & 2.00 & 3.00\\ 0.00 & 1.00 & 0.00 & 3.74 & 2.64 & 1.76\\ 5.12 & 4.37 & 5.39 & 2.36 & 4.66 & 2.45\\ 5.16 & 5.37 & 3.27 & 5.36 & 3.15 & 6.36\\ 4.46 & 3.26 & 6.29 & 6.24 & 2.12 & 5.62\\ 5.35 & 3.73 & 2.22 & 2.21 & 1.43 & 2.72 \end{array}\right] \end{array}$$ (30)

After normalization, the independent variable matrix and the dependent variable matrix are obtained as Eqs. (31) and (32):


$$\begin{array}{c} {X_{i}}^{{\;}^{\prime }}=\left[\begin{array}{c} X_{\text{1}}\\ X_{\text{2}}\\ X_{\text{3}}\\ X_{\text{4}}\\ X_{\text{5}}\\ X_{\text{6}} \end{array}\right]=\left[\begin{array}{cccccc} 0.00 & 0.02 & 0.075 & 0.032 & 0.131 & 0.393\\ 0.00 & 0.262 & 0.00 & 0.437 & 0.319 & 0.296\\ 0.736 & 0.663 & 0.836 & 0.466 & 0.585 & 0.295\\ 0.558 & 0.808 & 0.467 & 0.872 & 0.297 & 0.741\\ 0.61 & 0.296 & 0.85 & 1 & 0.264 & 0.539\\ 0.085 & 0.043 & 0.029 & 0.028 & 0.187 & 0.186 \end{array}\right] \end{array}$$ (31)

$$\begin{array}{c} {Y_{i}}^{{\;}^{\prime }}=\left[\begin{array}{c} {Y_{\text{1}}}^{{\;}^{\prime }}\\ {Y_{\text{2}}}^{{\;}^{\prime }}\\ {Y_{\text{3}}}^{{\;}^{\prime }}\\ {Y_{\text{4}}}^{{\;}^{\prime }}\\ {Y_{\text{5}}}^{{\;}^{\prime }}\\ {Y_{\text{6}}}^{{\;}^{\prime }} \end{array}\right]=\left[\begin{array}{cccccc} 0.089 & 0.193 & 0.539 & 1 & 0.193 & 0.193\\ 0.089 & 0.193 & 0.00 & 0.379 & 0.253 & 0.142\\ 0.185 & 0.446 & 0.727 & 0.336 & 0.467 & 0.255\\ 0.155 & 0.582 & 0.428 & 0.631 & 0.209 & 0.666\\ 0.529 & 0.290 & 0.680 & 0.709 & 0.306 & 0.589\\ 0.049 & 0.013 & 0.013 & 0.011 & 0.025 & 0.13 \end{array}\right] \end{array}$$ (32)

This leads to the difference matrix D:


$$\begin{array}{c} D=\left[\begin{array}{cccccc} 1.00 & 5.00 & 4.00 & 7.00 & 2.00 & 3.00\\ 2.00 & 2.00 & 0.00 & 3.58 & 3.04 & 2.56\\ 2.22 & 5.67 & 7.89 & 8.56 & 5.76 & 2.05\\ 2.16 & 2.87 & 3.27 & 7.96 & 3.15 & 6.96\\ 4.56 & 1.56 & 6.39 & 6.84 & 8.72 & 6.22\\ 6.25 & 3.13 & 0.22 & 5.81 & 3.43 & 4.02 \end{array}\right] \end{array}$$ (33)

From these results, the maximum and minimum difference values are determined as Dmax = 8.56 and Dmin = 0.00. Then η_ij_ can be calculated, which is substituted into Eq. (18) to obtain the grey correlation degrees of the six influencing factors.


$$\begin{array}{c} \lambda_{\text{1}}={\left[\begin{array}{cccccc} 0.60 & 0.57 & 0.67 & 0.61 & 0.56 & 0.63 \end{array}\right]}^{T} \end{array}$$ (34)

The results show that the influence of different factors on the porosity of the stabilized sulfate saline soil varies slightly. Among them, the influence of soil particles on the porosity of stabilized sulfate saline soil is relatively large, followed by salt content, calcium carbide slag particles and slag particles, whereas the influence of fly ash particles on the soil porosity is relatively small. The grey relational degree values of each factor are relatively close, indicating that the type of soil particles, curing age, and salt content all have significant influences on the porosity of the stabilized sulfate saline soil. This suggests that the impact of a single factor should not be considered in isolation, but rather the combined effect of multiple factors should be taken into account. The relational degree of soil particles is slightly higher than that of other factors, mainly because soil particles have the largest proportion and form the basis of the stabilized soil's skeleton structure. The relational degree of salt content is slightly higher than that of other particle types and curing age. This is because the salts in the sulfate saline soil undergo hydration reactions during the stabilization process, generating products such as gel and ettringite, which in turn change the internal pore structure of the soil. When the salt content is higher, the hydration reaction is more intense, and the variation in porosity is greater. The relational degree of curing age is the next highest. As the curing time increases, the hydration reaction of the stabilized soil gradually completes, and the hydration products continuously fill the soil pores, causing the porosity to continuously decrease. However, this decreasing trend will gradually level off in the later stage. The relational degrees of particle types such as electrostone slag and slag are relatively low, mainly because the physical forms and chemical activities of different particles vary, and their influence on porosity is more reflected in the optimization of the microstructure.

**Fig 7:**
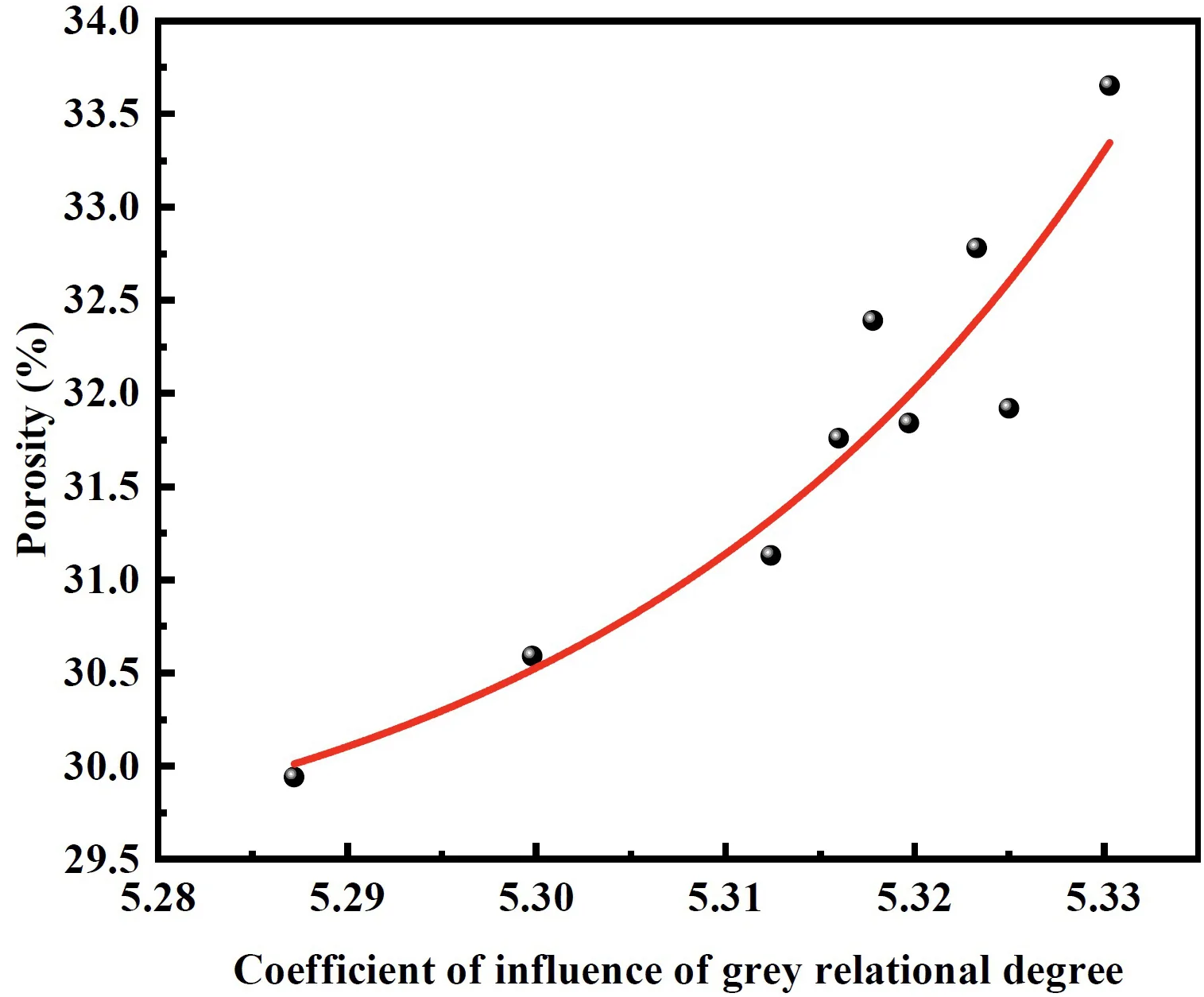
Fitting results of porosity and influencing factors.


By fitting the experimental data, the relationship between porosity and influencing factors can be derived. The fitting results are shown in Fig 7 and Eq. (35):

$$\begin{array}{c} N=29.1+0.058e^{\left(37.03^{\left(0.63d\sum_{i=1}^{\text{5}}A_{i}a_{i}\right)}-192.96\right)}{\text{\textasciitilde{}R}}^{2}=\text{\textasciitilde{}0.982} \end{array}$$ (35)

Where N is the porosity percentage (%); a_i_ is the grey correlation degree of the factors influencing porosity; ai is the content of the influencing factors (%); D, F, G, K, H, and L are fitting parameters. F is the rate of change under different influencing factor systems, G is the speed at which porosity changes with the coefficient of the particle system, D and L is a constant, K represents the grey correlation degree of the curing age, and H represents the number of influencing factors, and d is the curing age.

By using the least squares method combined with statistical tests, the fitted values, standard errors and 95% confidence intervals of each parameter in Eq (35) were obtained, as shown in Table 5 below:

**Table 5:** Parameter fitting result statistics.

Parameter	Estimated Value	Standard Error	95% Confidence Interval	t-value
**D**	29.1	0.21	[28.68, 29.52]	138.57
**F**	0.058	0.003	[0.052, 0.064]	19.33
**G**	37.03	1.25	[34.53, 39.53]	29.62
**K**	0.63	0.05	[0.53, 0.73]	12.60
**H**	5	0.32	[4.36, 5.64]	15.63
**L**	−192.96	8.74	[-209.94, -175.98]	−22.08

The goodness-of-fit indicators of the formula are as follows: the coefficient of determination R^2^ = 0.982, the root mean square error (RMSE) = 0.12%, the mean absolute error (MAE) = 0.09%, indicating that this formula has a high prediction accuracy for porosity.

#### 4.1.3 Equation for water migration in sulfate saline soil stabilized by all-solid-waste cementitious materials.

First, the L model based on the Logistic curve was applied to fit the measured soil-water characteristic curve data. The L model is expressed as follows:


$$\begin{array}{c} \theta =\frac{\theta_{s}}{1+ae^{blg\left(\varphi_{m}\right)}} \end{array}$$ (36)

Where θ is the volumetric water content, %; θ_s_ is the saturated water content, %; m is matric suction; a and b are parameters of the L model. The formula for m is derived as follows, according to Eq. (36):


$$\begin{array}{c} \varphi_{m}=10^{\frac{ln\left(\frac{\frac{\theta s}{\theta }-1}{a}\right)}{b}} \end{array}$$ (37)

**Fig 8:**
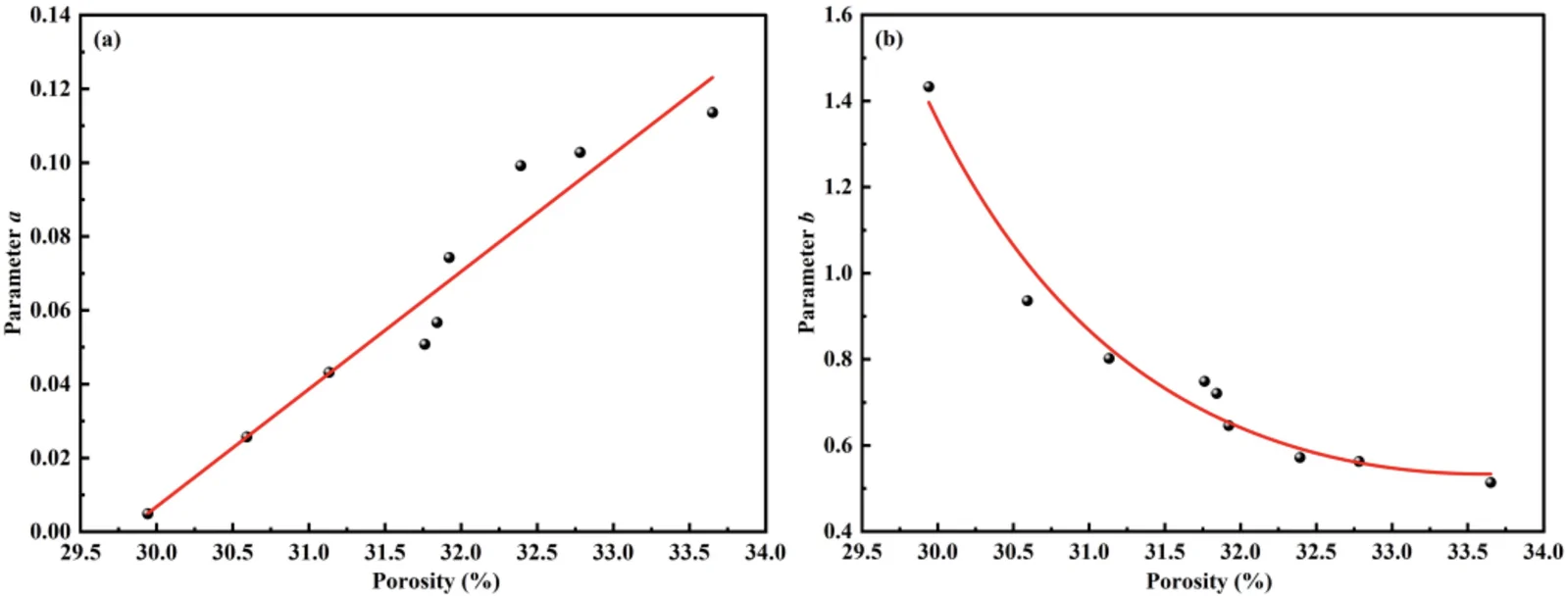
Prediction curves under different porosities. (a) Variation of parameter a with porosity, (b) variation of parameter b with porosity.

The fitting of the soil-water characteristic curve data was affected by the expansion porosity, and the fitting parameters are shown in Table 6. Moreover, it can be seen from Fig 8 that the parameters a and b of the soil-water characteristic curve of the full solid waste cementitious material solidified sulfate saline soil based on the porosity influence conform to the polynomial function relationship. The reason for choosing the polynomial function is that it can better fit the complex nonlinear relationship. In many soil property studies, the polynomial function has been successfully applied to describe similar correlations [46]. Through comparison with relevant theoretical models and existing research data, it is found that the polynomial function has high applicability in this research context [47].

**Table 6:** Parameters of L model fitting.

Porosity Parameter	a	B	$\theta_{s}$
$N=29.1+0.058e^{\left(37.03^{\left(0.63d\sum_{i=1}^{\text{5}}A_{i}a_{i}\right)}-192.96\right)}$	0.0049	1.433	44.235
	0.0257	0.936	46.168
	0.0432	0.802	48.414
	0.0508	0.749	49.225
	0.0567	0.721	49.999
	0.0743	0.646	51.428
	0.0992	0.572	53.616
	0.1028	0.563	54.432
	0.1136	0.514	56.303
**95% Confidence Interval**	[0.0293, 0.0931]	[0.532, 1.022]	[47.728, 54.902]

As shown in Fig 8, parameters a and b of the soil-water characteristic curve for sulfate saline soil stabilized with all-solid-waste cementitious materials exhibit a polynomial relationship with porosity. The specific prediction formulas are given below, where x denotes porosity:


$$\begin{array}{c} a=-0.9484+0.0318x{\text{\textasciitilde{}(R}}^{2}=0.9643) \end{array}$$ (38)

$$\begin{array}{c} b=e^{\left(80.5356-4.831x+0.0719x^{\text{2}}\right)}{\text{\textasciitilde{}(R}}^{2}=0.9620) \end{array}$$ (39)

The R^2^ value is close to 1, which indicates that the model fits the data well.

Substituting Eqs. (38) and (39) into Eq. (27) yields the calculation model for the soil-water characteristic curve considering the effect of porosity:


$$\begin{array}{c} \varphi_{m}=10\times e^{\frac{ln\left(\frac{\frac{\theta s}{\theta }-1}{-0.9484+0.0318x}\right)}{{\;}^{\left(80.5356-4.831x+0.0719x^{\text{2}}\right)}}} \end{array}$$ (40)

If we represent 10 to the power of γ, then:


$$\begin{array}{c} \gamma =10\times e^{\frac{ln\left(\frac{\frac{\theta s}{\theta }-1}{-0.9484+0.0318x}\right)}{{\;}^{\left(80.5356-4.831x+0.0719x^{\text{2}}\right)}}} \end{array}$$ (41)

$$\begin{array}{c} \varphi_{m}=10^{\gamma } \end{array}$$ (42)

Substituting Eq. (42) into Eq. (41), the total soil water potential can be expressed as:


$$\begin{array}{c} \varphi =z+10^{\gamma } \end{array}$$ (43)

Substituting Eq. (43) into Eq. (13) yields the prediction model based on the influence of porosity and water migration:


$$\begin{array}{c} \frac{\partial \theta }{\partial t}=k\frac{\partial^{\text{2}}(z+10^{\gamma })}{\partial z^{\text{2}}} \end{array}$$ (44)

#### 4.1.4 Equation for salt migration in sulfate saline soil stabilized with all-solid-waste cementitious materials.

Substituting Eqs. (18) and (43) into Eq. (17) yields the salt concentration control equation:


$$\begin{array}{c} \frac{\partial \left(\theta c\right)}{\partial t}=\frac{\partial }{\partial z}\left[kc\frac{\partial }{\partial z}\left(z+10^{\gamma }\right)\right]+\frac{\partial }{\partial z}\left(D_{sh}\frac{\partial c}{\partial z}\right) \end{array}$$ (45)

### 4.2 Verification of the water-salt migration model

The finite volume method was employed to solve the water-salt transport equation of the stabilized sulfate saline soil. A linear equation system was constructed in a discrete form. After iteration, the obtained solution approximates the original partial differential equation. A comparison of the numerical solution with the experimental results is shown in the figure below.

#### 4.2.1 Comparison of measured and calculated values for moisture migration.

**Fig 9:**
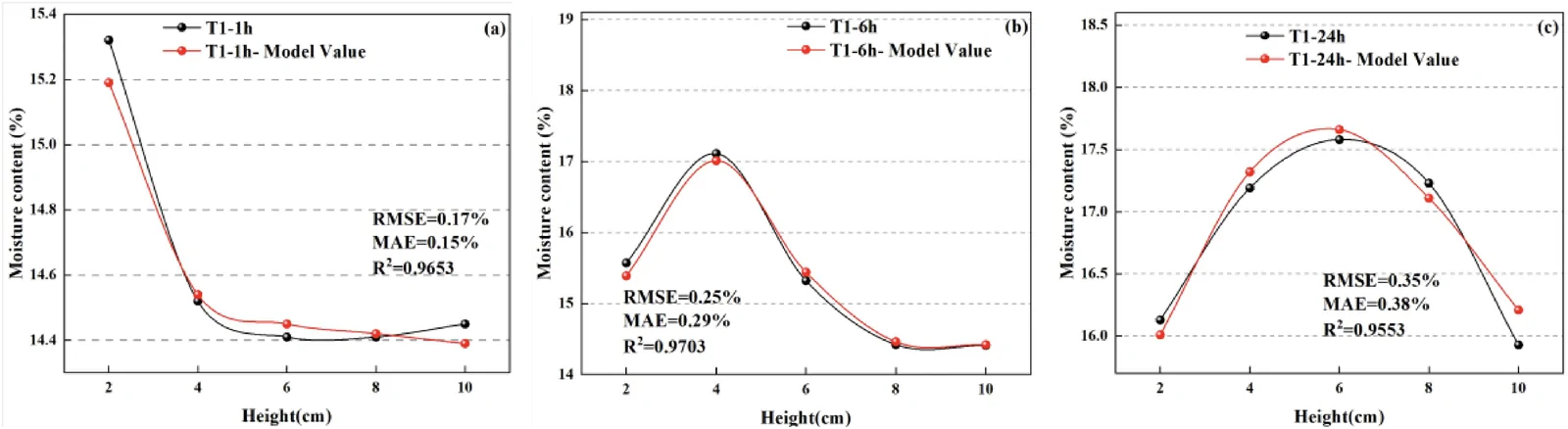
Measured and calculated values for Y1 moisture migration: (a) 1 h, (b) 6 h, (c) 24 h.

Fig 9 compares the measured values with the calculated values for specimen Y1. The results demonstrate that the model accurately captures the moisture migration pattern.

Within the first hour, the moisture level rose by approximately 6 cm. At this point, the measured moisture content at the bottom and the calculated value differed by 1.26%. After 24 hours, both the measured and calculated moisture levels reached the top of the specimen, with the measured value being slightly higher than the calculated one. The largest deviation occurred at 10 cm, where the error reached 3.99%. However, this remained within the allowable error margin of 15%.

**Fig 10:**
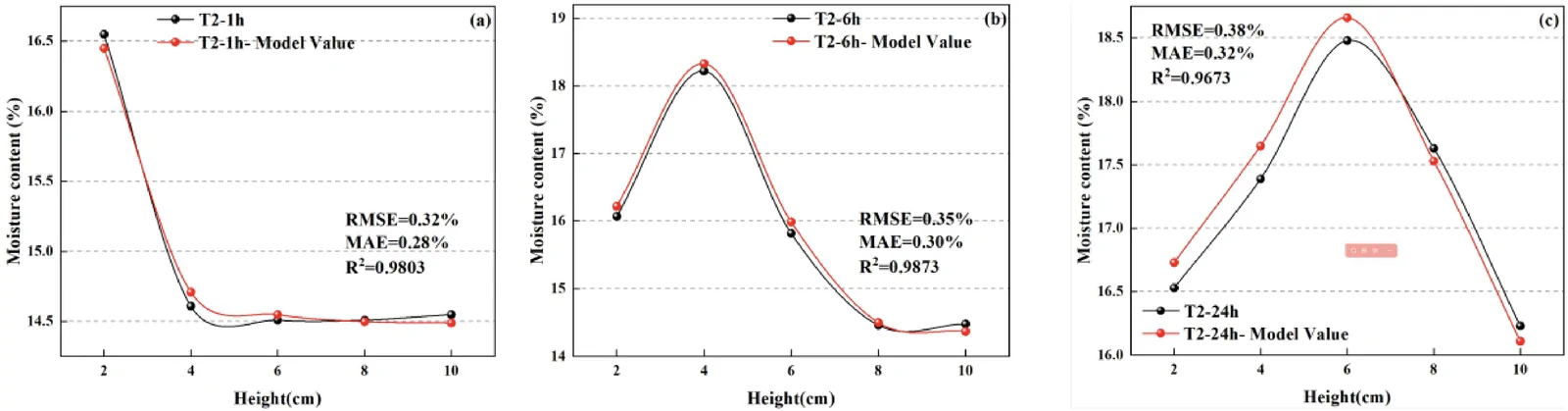
Measured and calculated values for Y2 moisture migration: (a) 1 h, (b) 6 h, (c) 24 h.

Fig 10 presents the comparison between the experimental values and the simulation predictions of water migration for specimen Y2 at various time points.

At 1 h, the calculated and measured water contents of Y2 at various heights were generally consistent. The maximum error of 3.85% occurred at 10 cm; at 6 h, water accumulated at 4 cm, and the error value at this point was 1.13%; at 24 h, the calculated value at 6 cm was slightly greater than the measured value, with the maximum error reaching 6.38%. Overall, the maximum error range of water migration for Y2 across different time periods was 1–5%.

In conclusion, RMSE ≤ 0.35%, MAE ≤ 0.38%, R^2^ ≥ 0.9553, indicating that the average deviation between the calculated values and the measured values of the model is low, the fitting degree is good, and the model has a high prediction accuracy for the law of water content variation with height.

#### 4.2.2 Comparison of measured and calculated values for salt migration.

**Fig 11:**
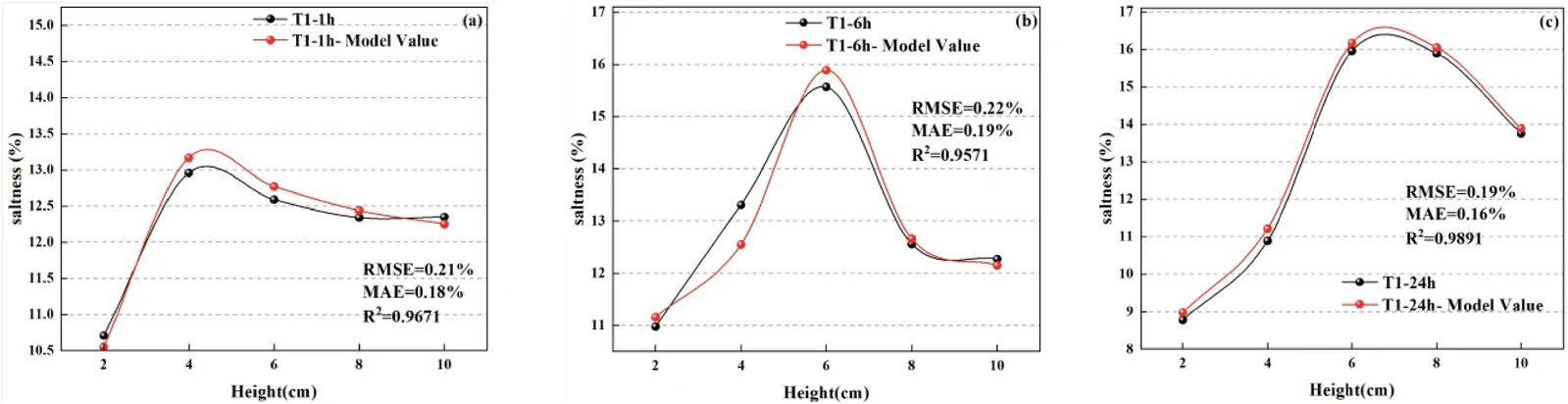
Measured and calculated values for Y1 salt migration: (a) 1 h, (b) 6 h, (c) 24 h.

Fig 11 compares the measured and calculated salt migration values for Y1 at different time periods. The two set of results show a high degree of consistency. At 1 h, the maximum error occurred at a height of 4 cm, with a value of 1.33%; at 6 h, the maximum error reached 7.83%; at 24 h, the measured salt content at 4 cm was slightly higher than the calculated value, with an error of 5.65%. All results were within the allowable error range of 15%.

**Fig 12:**
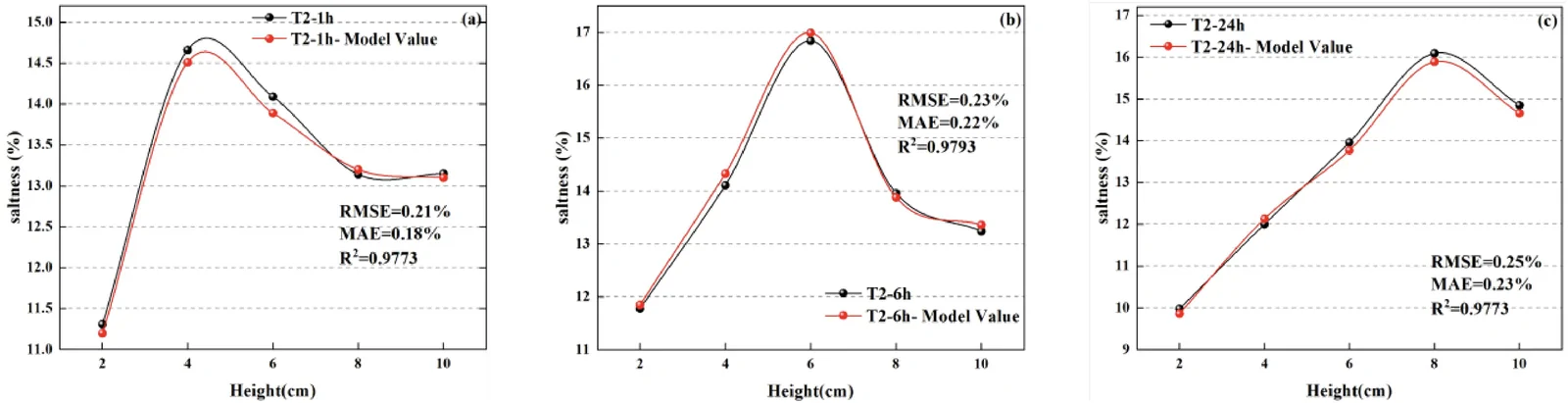
Measured and calculated values for Y2 salt migration: (a) 1 h, (b) 6 h, (c) 24 h.

Fig 12 compares the measured and calculated salt migration values for Y2 at different time periods. At 1 h, the maximum error occurred at a height of 6 cm, with a value of 1.67%; at 6 h, due to iterative errors, the calculated values at 3–5 cm were slightly greater than the experimental values, with a maximum error of 1.55%; at 24 h, the calculated salt content at 8 cm exceeded the measured value slightly, with the maximum error reaching 1.86%. Overall, the maximum error of salt migration for Y2 across different time intervals ranged within 1–2%.

The comparison results calculated and experimental values of water-salt migration in stabilized sulfate saline soil are highly consistent across different curing periods and cementitious material compositions. Errors associated with salt migration were slightly lesser than those for water migration. For water migration: the error value for Y1 was 3.99%, compared with 5.65% for Y2; for salt migration: Y1 showed errors of 1.13–6.38%, while Y2 showed errors of 1.55–1.86%. All errors remained within the allowable limit of 15%.

In conclusion, RMSE ≤ 0.25%, MAE ≤ 0.23%, R^2^ ≥ 0.9571, indicating that the average deviation between the calculated values and the measured values of the model is low, the fitting degree is good, and the model has a high prediction accuracy for the variation pattern of salt content with altitude.

## 5. Discussion

The established model for water-salt migration demonstrates good accuracy and applicability. In this section, the model is used to calculate and analyze: (i) the applicability of the water-salt migration model, (ii) Analysis of water and salt migration behavior based on cementitious material compositions, and (iii) the analysis of Water and Salt Migration Characteristics with Porosity

### 5.1 Applicability analysis of water-salt migration model

**Fig 13:**
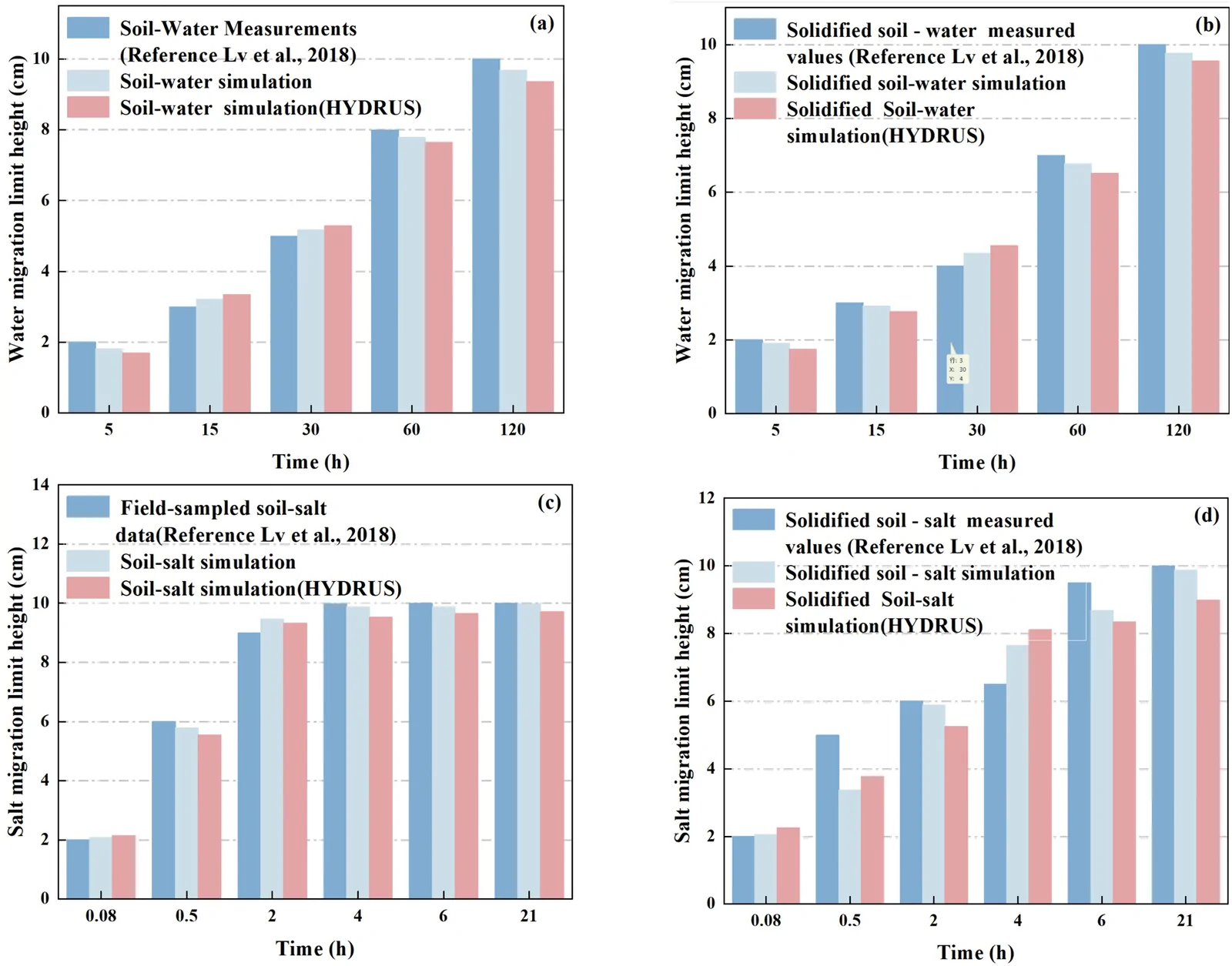
Comparison of calculated and measured values of the maximum migration height for water and salt: (a) water migration in saline soil, (b) water migration in stabilized sulfate saline soil, (c) salt migration in saline soil, (d) salt migration in stabilized sulfate saline soil.

Lv et al. [48] conducted a systematic study on the distribution of water-salt migration in stabilized sulfate saline soil. By comparing migration behavior in untreated saline soil and stabilized sulfate saline soil, they proved that stabilization can effectively inhibit water and salt transport. To further verify the applicability of the proposed model, water-salt migration results for both stabilized sulfate saline soil and untreated saline soil (as reported in Reference 35) were calculated using the established model. The calculation results are shown in Fig 13. For untreated saline soil, the model predicted migration heights of 9.68 cm (water) and 9.98 cm (salt) within 24 h, with errors of only 3.2% and 0.2%, respectively, compared with the measured values of 10 cm. For stabilized sulfate saline soil, the predicted migration heights were 9.77 cm (water) and 9.88 cm (salt), with corresponding errors 2.3% and 1.22%. The predicted 24-hour water and salt migration heights by the traditional model (HYDRUS model) were 9.36 cm and 9.72 cm respectively, with errors of only 6.4% and 0.28% compared to the measured values (10 cm); the predicted results for the compacted soil (9.56 cm and 8.99 cm) had errors of 4.4% and 10.01% respectively compared to the measured values. Compared with the 4%−11% error range typically reported for traditional models (HYDRUS) under similar conditions, the proposed model exhibits higher accuracy, further verifying its broader applicability.

In summary, the water-salt migration model developed in this study—by coupling the pore porosity-saturation relationship in this paper—represents an improvement over traditional approaches. It is not only suitable for stabilized sulfate saline soil but also demonstrates strong predictive performance for ordinary saline soil. By introducing pore porosity, the model overcomes the limitations of traditional models that struggle to represent the dynamic influence of material hydration products. It provides a reliable tool for predicting water-salt migration under varying soil and binder conditions.

### 5.2 Analysis of water and salt migration behavior based on cementitious material compositions

**Fig 14:**
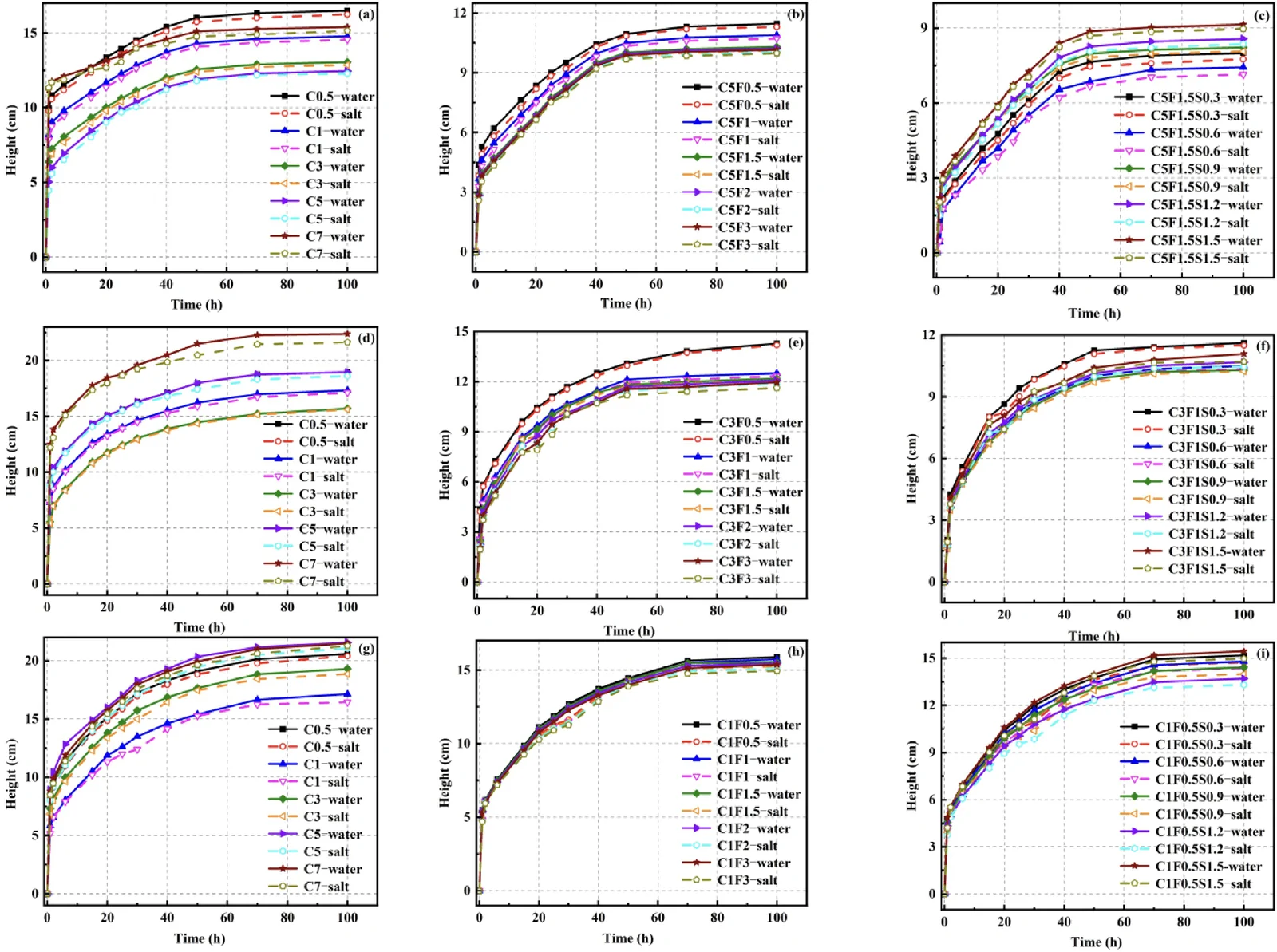
Water and salt migration under different cementitious material compositions: (a) 1% salt-CS, (b) 1% salt-CS-F, (c) 1% salt-CS-F-S,(d) 2% salt-CS, (e) 2% salt-CS-F, (f) 2% salt-CS-F-S,(g) 5% salt-CS, (h) 5% salt-CS-F, (i) 5% salt-CS-F-S.

Sulfate saline soils with salt contents of 1%, 2%, and 5% were selected to analyze the influence of cementitious material composition on water-salt migration. Using the established model, the migration heights of water and salt under representative combinations of cementitious materials were calculated, as shown in Fig 14.

From Figs 14(a), 14(d), and 14(g), it can be observed that with the single addition of calcium carbide slag (C), the water and salt migration heights of the specimens stabilized at approximately 50 h. Across different salt contents, the maximum migration heights of water and salt first decreased and then increased as the C content varied. When the C dosage was 5%, 3%, and 1%, the lowest migration heights were obtained, ranging within 12.86–17.13 cm. As shown in Figs 14(b), 14(e), and 14(h), under the optimal C dosage, when the F dosages were 1.5%, 1%, and 3% respectively, the maximum migration heights of water and salt were the lowest. Moreover, the migration heights of all specimens stabilized at around 60 h. As F dosage increased, the maximum migration heights of water and salt gradually approached stability, raging within 10.02–15.88 cm. As illustrated in Figs 14(c), 14(f), and 14(i), under the optimal C and F dosages, the maximum migration height followed a pattern of first decreasing and then increasing with increasing slag (S) dosage. At S dosages of 0.6%, 0.9%, and 0.9%, the maximum migration heights plunged, falling within 7.14–13.7 cm. The water and salt migration heights of each specimen stabilized at approximately 70 h.

In summary, the overall maximum migration heights of water and salt in the C–F–S system were markedly lower than those in the C–F system and the single C system, indicating that the C–F–S system provided the most effective stabilization. Within the same system, increasing salt content raised the maximum migration heights of water and salt by 4.1 cm and 4.17 cm, respectively, and extended the equilibrium time by 20 h-40 h. According to Chang [49], the best stabilization effect was achieved when using fly ash and calcium carbide slag in a dosage of 21%. The water and salt migration height reached 10 cm after 24 h. Therefore, the recommended dosages of cementitious materials are as follows. Low-salinity soils: calcium carbide slag (C) 3–5%, fly ash (F) 1.5–2%, slag (S) 0.3–0.6%. Medium-salinity soils: calcium carbide slag (C) 1–3%, fly ash (F) 1–1.5%, slag (S) 0.9–1.2%. High-salinity soils: calcium carbide slag (C) 1–3%, fly ash (F) 0.3–0.6%, slag (S) 0.9–1.2%. However, the recommended dosage mentioned above is a preliminary conclusion based on the limited experimental conditions and short-term test results of this study. Due to the fact that the engineering characteristics of sulfate-stained soil are influenced by various complex factors such as location, formation, and environment, before the actual engineering application, targeted tests should be conducted on the soil samples of specific projects. Combined with the on-site construction conditions and long-term performance monitoring data, the dosage should be further optimized and adjusted to ensure the reliability and stability of the solidification effect.

### 5.3 Analysis of water and salt migration characteristics with porosity

**Fig 15:**
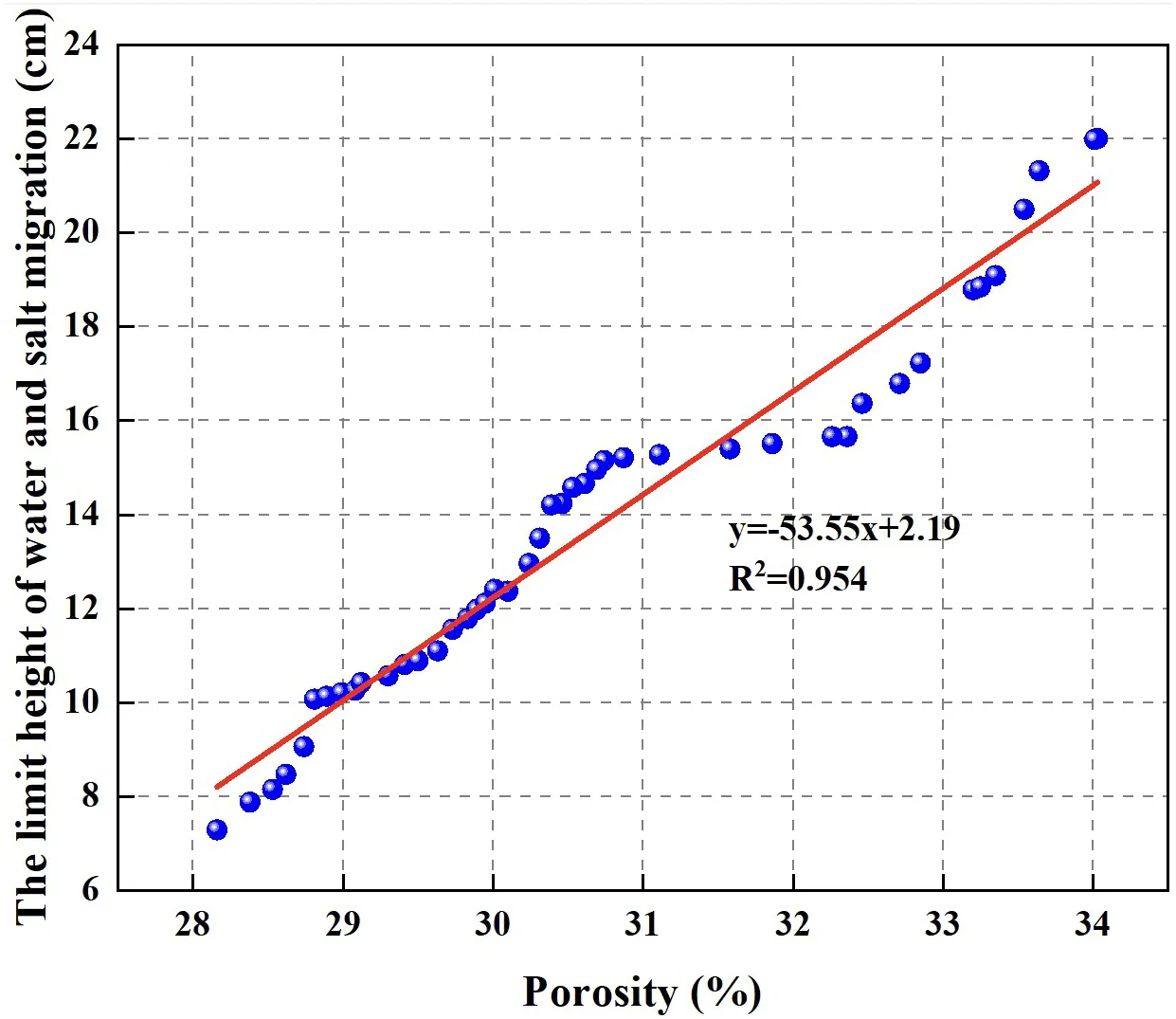
The relationship between porosity and the maximum height of water and salt migration.

To analyze the influence of pore volume on the water and salt migration in the solidified sulfate-stained soil. Using the established model, the migration heights of water and salt under different pore volumes were calculated as shown in Fig 15. The calculation results indicate that the pore volume has a decisive impact on the water and salt migration behavior of the fully solidified waste cementitious material for the sulfate-stained soil.

As the porosity increases, the maximum height of water-salt migration shows a linear growth trend (Fig 15). This pattern indicates that the increase in internal pores in the soil promotes the formation of capillary channels, resulting in an increase in the maximum height of water and salt migration. The porosity has a crucial controlling effect on the maximum height of water-salt migration. The dynamic evolution of porosity is closely related to the matrix suction and is a core coupling variable in the water-salt migration model. The water-salt migration model based on porosity (Eq. 40–45) further shows that changes in porosity will have an impact on the water-salt migration process and can be regarded as one of the important factors driving water-salt migration.

In conclusion, porosity, as the key controlling parameter for water and salt migration in solidified sulfate-stained soil, affects the maximum height of water and salt migration. This study has established a quantitative relationship between porosity and water and salt migration, providing a theoretical basis for the optimization design of salt-stained soil solidification projects.

## 6. Conclusions

This study investigated the effects of salt content, cementitious material dosage, and mix proportions of all-solid-waste cementitious materials on the stabilization of sulfate saline soil. Through water and salt migration tests, key parameters such as matric suction, moisture content, porosity, and salt content were examined to analyze the behavior of water and salt migration. Porosity was then introduced to characterize the time-dependent changes in the hydration products of cementitious materials. A logistic curve was applied to establish the relationship between porosity and matric suction, which served as the coupling link in the proposed water–salt migration model. The main conclusions are as follows:

(1) Changes in porosity directly affect the spatiotemporal distribution of water and salt in stabilized sulfate saline soil. As the cementitious material dosage increases (from 2% to 8%), the porosity decreases from 33.65% to 29.94%, resulting in a 67.8% reduction in water migration. This demonstrates that the inhibitory effect on water and salt migration is significantly enhanced. The logistic curve-fitting equation of porosity-matrix suction relationship can reflect the influence of pore dynamic evolution on water and salt migration.
(2) The calculated model for water and salt migration in stabilized sulfate-stained soil has a high degree of accuracy and can describe the temporal and spatial distribution patterns of water and salt in the stabilized sulfate-stained soil. The maximum error between the measured and predicted values of water migration height in stabilized sulfate saline soil is 2.3%, while that for salt migration height is 1.22%.
(3) Grey relational analysis identifies the relative importance of influencing factors on porosity in the following descending order: soil particles > salt content > calcium carbide slag particles > slag particles > fly ash particles.
(4) The essence of the spatio-temporal distribution changes of water and salt in solidified sulfate saline soil under different cementitious systems is the dynamic evolution of the pore filling of the hydration reaction products of cementitious materials. Pore ratio is one of the important factors driving the migration of water and salt. The proposed calculation model can accurately reflect the spatio-temporal distribution changes of water and salt in solidified sulfate saline soil in cold and arid areas.
(5) By combining the dynamic evolution of porosity with the Logistic curve, a physical model for coupling water and salt migration was constructed. The grey correlation degree theory was used to quantify the influence weights of soil particles, salt content, and cementing materials on the pore structure. The model enables the improvement of saline soil at a low cost and with high performance.

## Supporting information

S1 Data Dataset. (XLSX)
